# Trends and causes of maternal death at the Lagos University teaching hospital, Lagos, Nigeria (2007–2019)

**DOI:** 10.1186/s12884-022-04649-4

**Published:** 2022-04-25

**Authors:** Joseph A. Olamijulo, Gbenga Olorunfemi, Halimat Okunola

**Affiliations:** 1 Department of Obstetrics and Gynecology, College of Medicine, University of Lagos, Lagos, Nigeria; 2 Department of Obstetrics and Gynecology, Lagos University Teaching Hospital, Lagos, Nigeria; 3 Division of Epidemiology and Biostatistics, School of Public Health, University of Witwatersrand, Johannesburg, South Africa

**Keywords:** Direct causes, Hypertension, Indirect causes, Join point trends, Lagos, Low- and middle-income countries, Maternal mortality, Nigeria, Sub-Saharan Africa

## Abstract

**Background:**

Nigeria has one of the worst global maternal mortality ratios (MMR). Institutional audit is invaluable in providing useful information for formulating preventive interventions.

**Objective:**

To evaluate the trends, causes and socio-demographic characteristics of maternal mortality at a tertiary institution in South-western Nigeria over a period of 13 years (2007–2019).

**Methodology:**

Cross sectional and temporal trend analysis of maternal deaths were conducted using chart reviews from 2007 to 2019. Socio-biological characteristics, booking status and ranking of clinical causes of maternal deaths were derived from patients’ chart review (2007–2019). Bivariate analysis and Annual percent change (APC) of the observed trends was respectively conducted using Stata version 17 and Joinpoint version 4.5.0.1 software respectively.

**Results:**

In the period 2007–2019, the mean age at maternal death was 30.8 ± 5.9 years and 88.8% of mortalities occurred among the “unbooked” women. The leading causes of direct maternal mortality were Hypertension (27.0%), Sepsis (20.6%) and haemorrhage (18.7%), while anaemia in pregnancy (3.2%), Human Immunodeficiency Virus (3.2%) and Sickle Cell Disease (2.4%) were the leading indirect causes of maternal mortality Joinpoint estimates showed a statistically significant increase in MMR of about 3.4% per annum from 2211 per 100,000 live births in 2007 to 3555.6 per 100,000 live births in 2019 (APC: +3.4%, *P*-value < 0.001).

**Conclusion:**

Contrary to some other reports, there was an increase in the institutional MMR between 2007 and 2019 even though the leading causes of death remained similar. Targeted interventions based on accurate data are urgently required in order to achieve the Sustainable Development Goal (SDG) 3.1.

**Supplementary Information:**

The online version contains supplementary material available at 10.1186/s12884-022-04649-4.

## Introduction

Maternal mortality is a major public health problem in the Low- and Middle-Income Countries (LMIC) such as Nigeria and it is one of the strongest indicators of a country’s standard of living and maternity care [1]. Globally, about 303,000 maternal deaths occurred in 2015 with 99% of these deaths occurring in LMICs [2]. The Sub-Saharan African countries (SSAs) had the greatest burden of maternal deaths as the regional maternal mortality ratio (MMR) in SSAs (546 deaths per 100,000 live birth) was about 45 times higher than the MMR in high income countries (HICs) (12 deaths per 100,000 live births) [2]. The disproportionate burden of maternal deaths in SSA illustrates the disparities in the burden of preventable socio-economic inadequacies and sub-optimal health systems between the HICs and the LMICs.

Most maternal deaths in the LMICs are preventable, as pregnancy and puerperium are largely physiological processes and the leading causes of maternal mortality are either preventable or treatable [2, 3]. Over the years, the eradication of the inequity and burden of global maternal mortality rates has been a major goal of the United Nations and global health and development organisations [2–4]. The 5th Millennium Development Goal (MDG 5) was aimed at reducing MMR by 75% by 2015 using the 1990 estimates as baseline [2, 5]. However, at the close of the MDGs in 2015, only 22 out of 181 countries achieved MDG5 [2]. Subsequently, in 2015 the Sustainable Development Goals (SDG) 3.1 aimed to reduce the national MMR to less than 70 per 100,000 (from the global MMR of over 500) by 2030 [2]. To achieve such target, it is imperative for countries to initiate, sustain and monitor useful interventions aimed at prevention of maternal mortality.

Nigeria is a Lower- Middle-Income country with an approximate population of 180 million [6]. The World Health Organisation (WHO) reported that about 58,000 maternal deaths occurred in Nigeria in 2015, and that the MMR declined from 1350 deaths per 100,000 live births in 1990 to 814 deaths per 100,000 live births in 2015 [2, 6]. The huge burden of maternal death in Nigeria is second to only India. The Nigerian MMR is also extremely high when compared to the MMR in HICs [2]. Hence, Nigeria must improve its preventive interventions to achieve the SDG target of less than70 deaths per 100,000 by 2030.

A major constraint to the development, implementation and monitoring of interventions aimed at combating the problem of maternal mortality in Nigeria is lack of reliable data, as there is no organised vital registration system in the country [3, 4, 6, 7]. Thus, the WHO estimates of maternal deaths in Nigeria may be inaccurate. A major source of information on maternal mortality trends in Nigeria is through review of hospital records [3, 6, 8]. Most of institutional trend studies on maternal mortality had hitherto utilised descriptive statistics [3, 4, 8–11]. Analytical trends techniques, as opposed to traditional descriptive approaches can help to refine conclusions from trends studies [3, 4, 8–11]. Therefore, the present study is aimed at determining the trends, causes and socio-demographic characteristics of maternal deaths at a tertiary hospital in South-western, Nigeria over a 13-year period (2007 to 2019), using Join point regression modelling. This will serve to improve conclusions and inform policy decision. The study will also provide a snapshot of our progress towards the realisation of the SDG 3.1.

## Methodology

This was a retrospective cross sectional and trends analysis study of all maternal deaths at the Lagos University Teaching Hospital (LUTH), a 761-bed tertiary hospital in South-western, Nigeria from 1st January 2007 to 31st December 2019. The hospital is one of the three public institutions providing tertiary obstetric care to the metropolitan city of Lagos and her environ. LUTH receives referrals from both government and private health facilities in Lagos state and adjoining states and conducts an average of 1500 deliveries per annum.

Maternal death is defined as the death of a woman while pregnant or within 42 days of delivery or termination, irrespective of the duration and site of the pregnancy from any cause related to or aggravated by the pregnancy or its management but not from accidental causes [2].

All maternal deaths in the hospital from 1st January 2007 to 31st December 2019 were identified by reviewing all the registers at the accident and emergency department, labour ward, theatre, antenatal and post-natal wards, intensive care unit and the mortuary department. Since every corpse was deposited in the mortuary, the mortuary record was used to confirm the list of maternal deaths obtained from other records ensuring that there was no omission nor duplication. The case files of the patients were retrieved from the medical records department and data on socio-demographic characteristics, clinical cause of deaths, highest cadre of doctor who physically attended to the patients and booking status were extracted using a standardized proforma. A booked patient is one who registered and was attending the antenatal clinic of the hospital prior to admission while an unbooked patient was one who did not register for antenatal care at our centre but whose emergency admission was her first attendance to the hospital via the emergency department. Annual live births were obtained from the birth register in the labour ward. The records of the departmental monthly maternal mortality reviews were also retrieved to ascertain the agreed cause of death.

### Death ascertainment

The department of obstetrics and Gynaecology conducts monthly maternal mortality review. At the meeting, a multidisciplinary team of obstetricians, anaesthetists, neonatologist and pathologists discuss each case of maternal mortality and a cause of death is determined and documented based on the input of all the team members. The agreed cause of death was utilised in this study. The 10th revision of the International Statistical Classification of Diseases and related problems (ICD-10) diagnostic codes by the World Health Organization (WHO) were used to classify the maternal deaths.

### Trends analysis from 2007 to 2019

The annual maternal deaths and maternal mortality ratio (maternal death / total deliveries or live births) was calculated from the chart review conducted between 2007 and 2019. The trends analysis of annual MMR between 2007 and 2019 was conducted using Joinpoint regression software (version 4.3.1). Poisson regression approach with a maximum of 2 join points was utilised and 4499 permutation tests were conducted for the trends [12, 13]. The average annual percent change (AAPC) of the trends (with its 95% confidence interval and *P*-value) was calculated. The MMR in 2015 was identified as an outlier and was excluded from the calculation of the trends analysis as it was extremely high and more than twice all other MMRs. A statistically significant positive or negative AAPC was respectively described as statistically significant increased or decreased trends. While APC between − 0.5 and + 0.5 with non-statistically significant *P*-value is assumed to be a stable trend.

The trend in the cause of maternal death was analysed by conducting annual ranking based on the proportion (%) of each cause of death. The causes of death were also classified into direct and indirect causes.

### Bivariate analysis of the socio-demographic characteristics and cause of death (2007–2019)

Data was entered on excel spreadsheet and then imported into STATA (Statacorp, Texas USA) version 16 statistical software. The socio-demographic and clinical characteristics of the decedents between 2007 and 2019 were described. Categorical variables were presented as frequency and percentages while continuous variables were presented as mean (±Standard Deviation) or median (interquartile range) based on test of normality. The association between socio-demographic and clinical characteristics and the three leading causes of direct maternal deaths (haemorrhage, hypertension, sepsis) was conducted using Pearson’s Chi-square test for categorical variable, while for continuous variables, analysis of variance (or Kruskal Wallis test) was utilised to test the differences in mean or median across groups. The data was further divided into two periods from 2007 to 2012 and 2013–2019 and compared.

Statistically significant level was set at a *P-*value < 0.05. Two-tailed test of hypothesis was assumed.

### Ethical considerations

Ethical approval for this study was obtained from the human research and ethics committee of the Lagos University Teaching Hospital, LUTH, Lagos (Ref. No: ADM/DCST/HREC/APP/3867). The study was strictly conducted according to international ethical principles, and we confirm that all methods were performed in accordance with the relevant guidelines and regulations of the ethics committee. The data was anonymised and there was no breach of confidentiality and autonomy of the deceased patients. The study was a retrospective study of routinely collected anonymised data and did not require obtaining informed consent from the decedents. Informed consent waiver was obtained from the human research and ethics committee of the Lagos University Teaching Hospital, LUTH, Lagos through ethics certificate with Ref. No: ADM/DCST/HREC/APP/3867.

## Results

### Trends in maternal mortality from 2007 to 2019

**Fig. 1:**
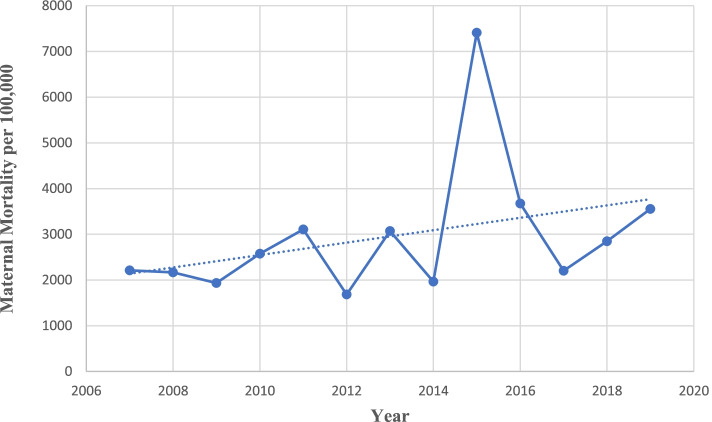
Trends in annual maternal mortality ratio in LUTH (2007–2019)

A total of 483 maternal deaths and 17,948 live births occurred from 2007 to 2019, giving a maternal mortality ratio of 2674.4 per 100,000 live births. Figure 1 showed the MMR between 2007 and 2019. Except for an extreme peak in 2015 at 7407/100,000 live birth, the MMR ranges between 1682/100,000 live births (2012) and 3556/100,000 live births (2019) (Additional file 1).

**Fig. 2:**
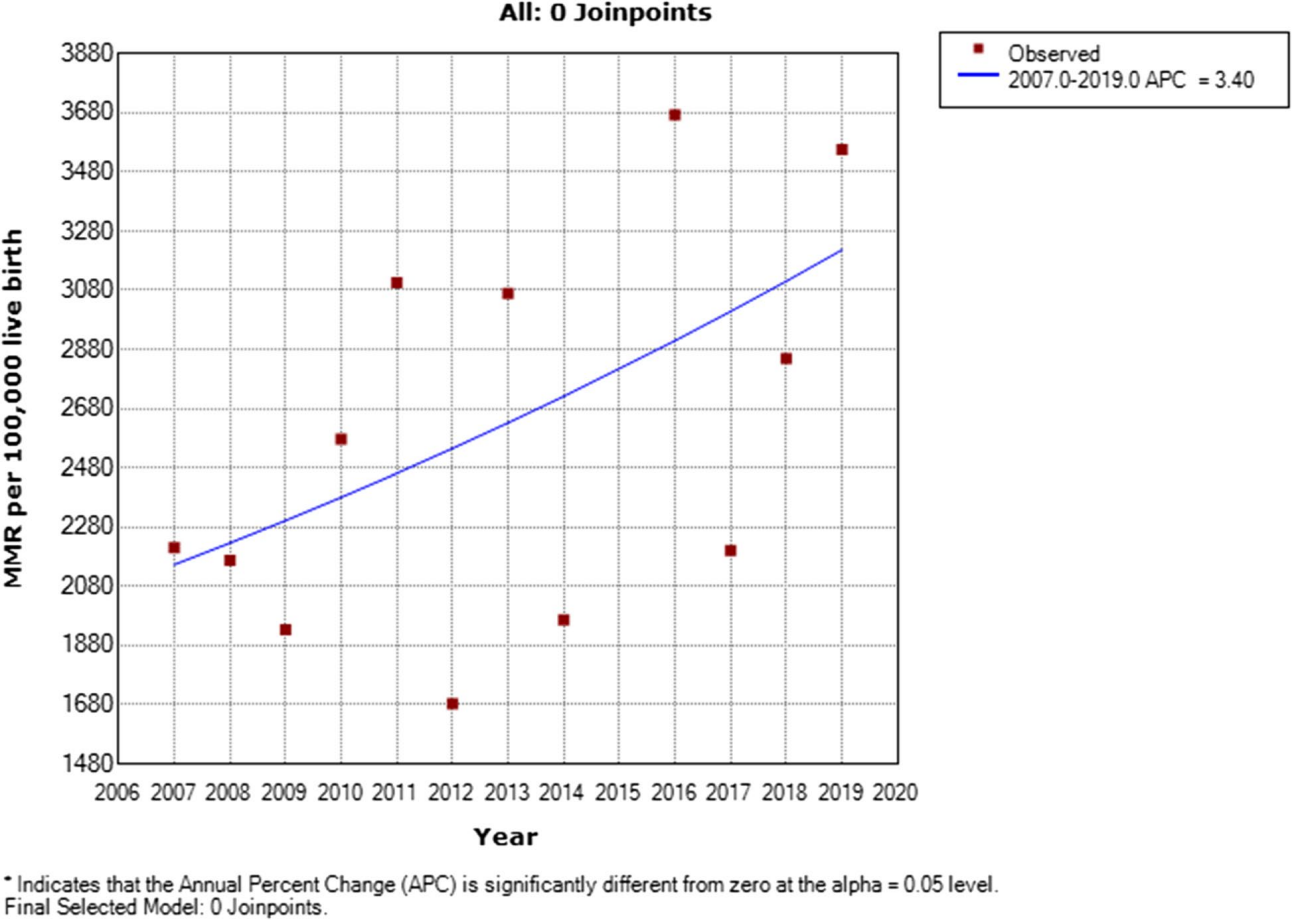
Joinpoint trends analysis with zero join point of annual maternal mortality ratio in LUTH (2007–2019)

Based on Joinpoint regression modelling and after excluding year 2015 being an outlier, there was an overall statistically significant increasing trend of MMR of about 3.9% per annum from 2007 to 2019. (AAPC = + 3.4%; 95%CI: − 0.3 to 7.3%, *P*-value = 0.10) Figure 2.

### Socio-biological and clinical characteristics of maternal deaths from 2007 to 2019

Table 1 shows that between 2007 and 2019, the mean age at maternal death was 30.9 ± 5.9 years and about half of the deceased were in the fourth decade of life (*n* = 262/483, 54.9 95%CI: 50.4–59.4%). The majority were married (84.1, 95%CI: 79.1–88.2%) and the majority were reviewed physically by a consultant obstetrician before death (73.0, 95%CI: 68.3–77.1%). More than a quarter of maternal deaths (*n* = 106/483, 28.9, 95%CI: 24.5–33.7%) occurred among nulliparous women. The median gestational age was 36(30–38) weeks and most of the mortalities occurred in the third trimester (*n* = 166/483, 82.2, 95%CI: 76.2–86.9%). More than four-fifths (88.8%) of maternal deaths occurred among unbooked patients.

**Table 1:** Socio-biological and clinical characteristics of maternal deaths from 2007 to 2019

Characteristics	Overall (2007–2019) ***n*** = 483, (%)	2007–2012 ***n*** = 256, (%)	2013–2019 ***n*** = 227, (%)	P-value
**Age (mean ± SD) years**	30.9 ± 5.9	30.3 ± 6.1	31.5 ± 5.5	0.0198*
< 20	13 (2.7)	10 (3.9)	3 (1.4)	0.054
20–29	166 (34.8)	98 (38.4)	68(30.6)	
30–39	262 (54.9)	127 (49.8)	135 (60.8)	
40–49	36 (7.6)	20 (7.8)	16 (7.2)	
**Marital status**				
Married	212 (84.1)	141 (80.6)	71 (92.2)	0.020*
Not married	40 (15.9)	34 (19.4)	6 (7.8)	
**Parity median (IQR)**	1 (0–3)	1 (0–3)	1(1–3)	0.3748
0	106 (28.9)	62 (33.3)	44 (24.3)	0.049*
1–4	244 (66.5)	119 (64.0)	125 (69.1)	
≥5	17 (4.6)	5 (2.7)	12 (6.6)	
**Gestational age**	36(30–38)	36(32–38)	35(28–38)	0.0467*
First trimester (< 14 weeks)	13 (6.4)	2 (2.9)	11(8.3)	0.005*
Second trimester (14–27 weeks)	23 (11.4)	2(2.9)	21 (15.8)	
Third trimester (≥ 28 weeks)	166 (82.2)	65 (94.2)	101(75.9)	
**Booking status**				
Booked	52 (11.2)	19 (7.6)	33 (15.4)	0.008*
Unbooked	411 (88.8)	230 (92.4)	181(84.6)	
**^Cadre of managing Doctor**				
Consultant	286 (73.0)	119 (63.0)	167 (83.5)	< 0.001*
Senior Registrar	96 (24.7)	64 (33.9)	32 (16.0)	
Registrar	7 (1.8)	6 (3.2)	1 (0.5)	
**ICU admission**				
Yes	126 (27.5)	80 (33.9)	46 (20.7)	0.002*
No	332 (72.5)	156 (66.1)	176 (79.3)	

*ICU* Intensive care Unit; * Statistically significant *P*-value < 0.05 ; ^ The most senior doctor who physically attended to the patient

### Comparison of the periods 2007–2012 and 2013–2019

The mean maternal age at death increased slightly from 30.3 ± 6.1 years in 2007–2012 to 31.5 ± 5.5 in 2013–2019 period (*P*-value = 0.0198) and 60.6% of deaths occurred in the fourth decade of life during 2013–2019 period as compared to 49.8% of maternal deaths that occurred in the fourth decade of life during the 2007–2012 period. There was increased proportion of maternal deaths in the first and second trimesters from 5.8% during 2007–2012 period to about 24.1% during the 2013–2019 period. Direct (physical) consultant involvement before death increased from 63.0% of cases during 2007–2012 to 83.5% during the 2013–2019 period but the prevalence of ICU admission decreased from 33.9 to 20.7% during the latter period (2013–2019) Table 1.

Table 2 shows that between 2007 and 2019, nearly two-thirds (64.0%) of maternal deaths were due to the three leading causes (Hypertension 27.4, 95% CI: 23.2–31.3%, Sepsis 20.6, 95% CI: 17.2–24.5% and Obstetric haemorrhage 18.7, 95% CI: 14.3–24.4%). While the proportion of maternal deaths from hypertension declined from 29.9% in 2007–2012 to 23.6% in 2013–2019 period, the prevalence of Obstetric hemorrhage and sepsis respectively increased from 14.9 and 18.1% in 2007–2012 period to 23.1 and 23.6% in the 2013–2019 period respectively. Anaemia in pregnancy (3.5%) and HIV (3.5%) were the leading indirect causes of maternal death followed by Sickle cell disease (2.4%) and Cardiac disease (2.2%).

**Table 2:** Clinical causes of maternal deaths in LUTH from 2007 to 2019

Direct causes	2007–2019 n, (%)	2007–2012 n, (%)	2013–2019 n, (%)	P-value
Hypertension	126 (27.0)	76 (29.9)	50 (23.6)	0.001^*^
Sepsis	96 (20.6)	46 (18.1)	50 (23.6)	
Post-partum Haemorrhage	69 (14.8)	28 (11.0)	41(19.3)	
Uterine Rupture	33 (7.1)	25 (9.8)	8 (3.8)	
Induced Abortion	31 (6.7)	24 (9.5)	7 (3.3)	
Antepartum Haemorrhage	18 (3.9)	10 (3.9)	8 (3.8)	
Ectopic Pregnancy	14 (3.0)	6 (2.4)	8(3.8)	
**Indirect Causes**				
Anaemia in pregnancy	15 (3.2)	7 (2.8)	8 (3.8)	0.481
^$^HIV	15 (3.2)	9 (3.5)	6 (2.8)	
Sickle cell disease	11 (2.4)	7 (2.8)	4 (1.9)	
Cardiac disease	10 (2.2)	7 (2.8)	3 (1.4)	
Anaesthetic	2 (0.4)	1(0.4)	1 (0.5)	
Diabetes Mellitus	2 (0.4)	1(0.4)	1 (0.5)	
Malaria	2 (0.4)	1(0.4)	1 (0.5)	
Unknown	6 (1.3)	2 (0.8)	4(1.9)	
^£^Others	20 (4.3)	6 (2.4)	14 (6.6)	

^$^*HIV* Human Immunodeficiency Syndrome; ^£^Others are causes that occurred with a frequency of 1; ^*^Statistically significant at *P*-value < 0.05

### Annual trends in causes of maternal mortality in LUTH, Lagos (2007–2019)

**Fig. 3:**
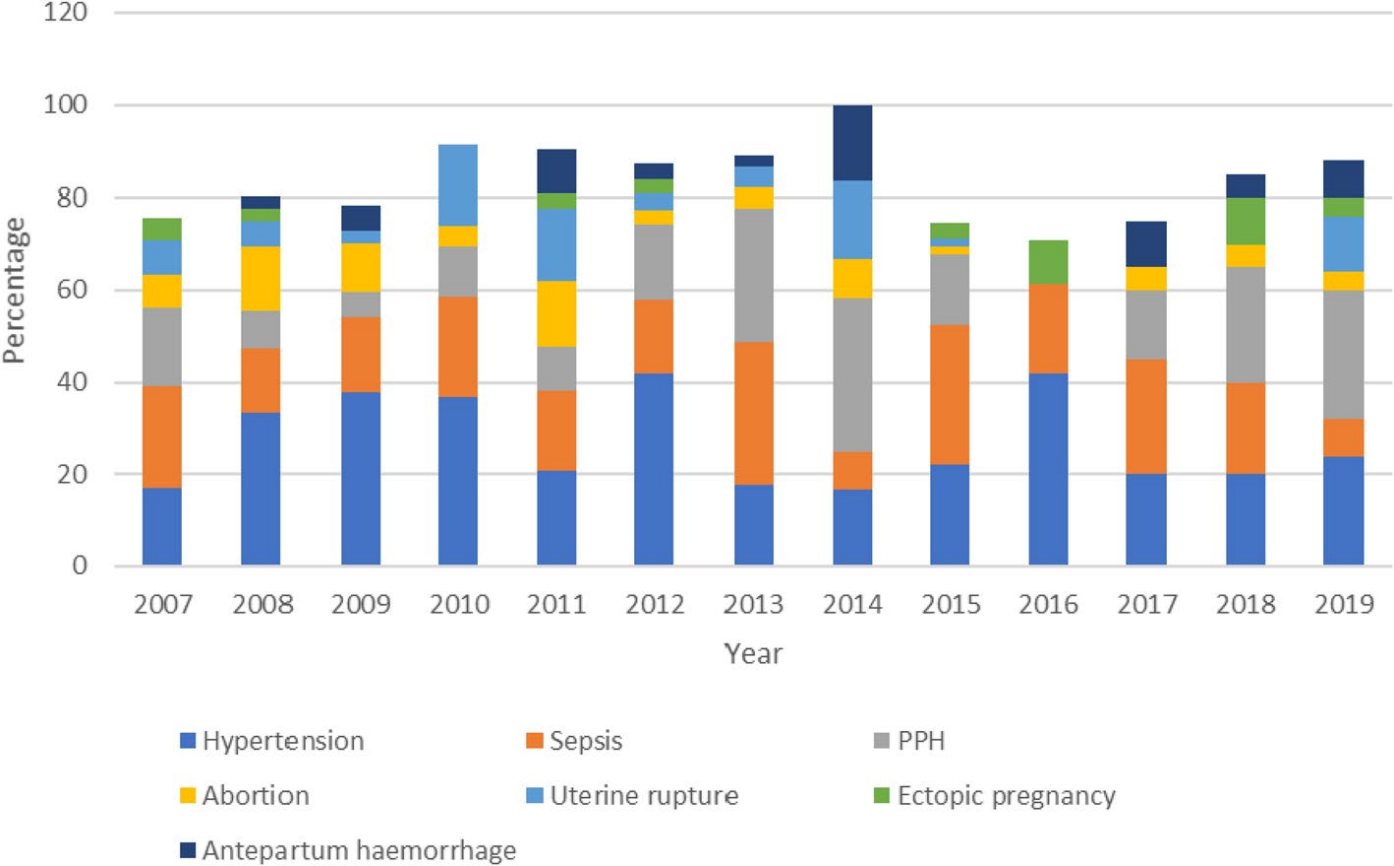
Annual trend of the direct causes of maternal death at LUTH, Lagos (2007–2019)

From Fig. 3, hypertensive disorders were the leading cause of maternal mortality from 2008 to 2012 and in 2016 while obstetric haemorrhage was the leading cause of annual maternal deaths in the latter period of the study (2013, 2014 and 2018–2019). No death was due to obstetric haemorrhage in 2016 for no obvious reason. However, haemorrhage contributed 15.3 and 15.0% to maternal deaths in 2015 and 2017 respectively. Sepsis was also a major cause of maternal death in the study period and it was the leading cause of maternal deaths in 2007, 2013, 2015 and 2017 respectively.

**Fig. 4:**
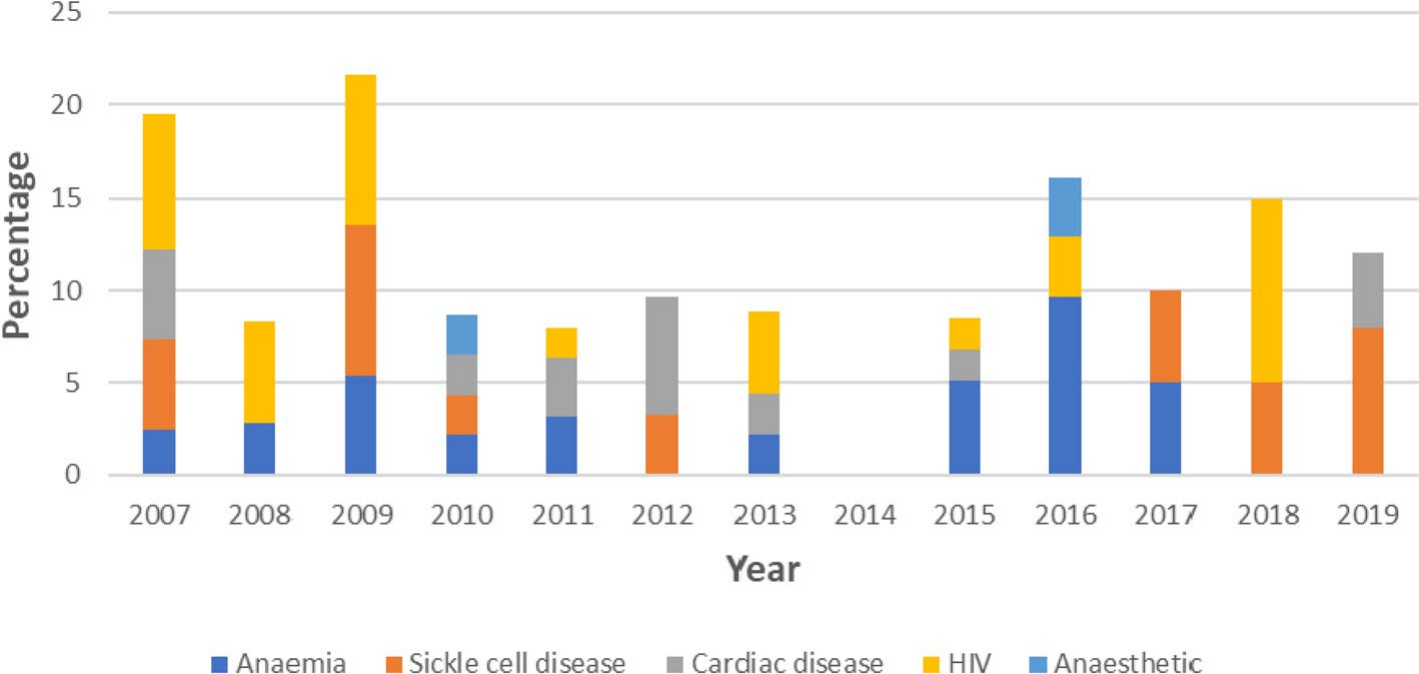
Annual Trend of the leading causes of indirect maternal death at LUTH, Lagos (2007–2019)

Figure 4 shows that Anaemia, HIV and Sickle cell disease were the leading indirect causes of maternal death in nearly all the years between 2007 and 2019. The proportion of maternal deaths due to HIV/AIDS fluctuated between 2007 and 2019 ranging between 1.6% in 2015 and 10% in 2018. However, HIV/AIDS did not contribute to maternal mortality in 2010, 2012,2014, 2017 and 2019. Notably, indirect causes did not contribute to maternal mortality in 2014.

Table 3 shows that abortion (40.0%) was the leading cause of death among teenagers followed by hypertensive disorders (26.7%) and sepsis (13.3%). However, sepsis was the leading cause of death among young women aged 20–29 years while hypertension was either the leading cause (30–39 years) or second leading cause (40 years and older) among older women. On the other hand, obstetric haemorrhage was the leading cause of death among women older than 40 years and second leading cause of death among women aged 30–39 years. All maternal deaths from cardiac disease, ectopic pregnancy, sickle cell disease and HIV occurred among women older than 30 years.

**Table 3:** Ranking of causes of maternal death by age category 2007–2019

Ranking	< 20 years	20–29 years	30–39 years	40 years and above
1	Abortion (40%)	Sepsis (24.6%)	Hypertension (30.5%)	Post-partum Haemorrhage (14.5%)/ Antepartum Haemorrhage (12.2%)
2	Hypertension (26.7%)	Hypertension (23.5%)	Sepsis (21.5)	Hypertension (24.4%)
3	Sepsis (13.3%)	Post-partum Haemorrhage (14.4%)/ Antepartum Haemorrhage (2.7%)	Post-partum Haemorrhage (15.9%)/ Antepartum Haemorrhage (3.1%)	Sepsis (17.1%)
4	–	Obstructed labour/uterine rupture (9.6%)	Obstructed labour/uterine rupture (6.2%)	Obstructed labour/uterine rupture (9.8%)
5	–	Abortion (9.1%)	Others (4.8%)	Cardiac (7.3%)
6	–	Others (4.8%)	Human immunodeficiency Virus (4.2%)	Others (7.3%)
7	–	–	Anaemia (3.1%)	Ectopic pregnancy (2.4%)
8	–	–	Abortion (2.8%)	Anaemia (2.4%)
9	–	–	Cardiac disease (2.4%)	Human immunodeficiency Virus (2.4%)
10	–	–	Ectopic pregnancy (2.4%)	Ectopic pregnancy (2.4%)
11		–	Anaesthetic (1.7%)	–
12		–	Sickle cell disease (1.4%)	–

### Socio-biological factors and maternal mortality

Table 4 shows the association between selected socio-biological factors and the leading causes of maternal mortality. Unbooked patients accounted for 88.8% (*n* = 411/463, 88.8%; 95%CI: 85.5–91.3%) of maternal deaths compared with booked patients that accounted for only 11.2%. Conversely, 19.8% of women that died of haemorrhage were booked patients while very few maternal deaths from hypertensive disorders (9.1%) and Sepsis (4.3%) occurred in women who booked for antenatal care in the hospital. About 95.7, 90.9 and 80.2% of deaths from sepsis, hypertensive disorders and haemorrhage respectively occurred in unbooked women. Although women who died from haemorrhage (32.2 ± 5.3 years) were older than women who died from hypertension (30.9 ± 5.5 years) or sepsis (30.4 ± 5.5 years), the differences did not reach statistical significance (*P*-value = 0.0685). However, there was a statistically significant difference in median parity among the three leading causes of maternal mortality as women who died of haemorrhage had higher median parity of 2 [1–4]) as compared to Hypertensive disorders (1 (0–3)) and Sepsis (1 (0–2)) (P-value =0.0253). Deceased women who had hypertensive disorder had the highest ICU admission rate (42.5%) followed by haemorrhage (20.2%) and Sepsis (19.6%).

**Table 4:** Association between socio-demographic factors and the leading causes of direct maternal deaths. (2007–2019)

Characteristics	Leading causes of maternal Deaths, ***n*** = 309			P-value
	Hypertension ***n*** = 126 (%)	Sepsis ***n*** = 96 (%)	Haemorrhage ***n*** = 87 (%)	
**Age (mean ± SD) years**	30.9 ± 5.5	30.4 ± 5.5	32.2 ± 5.3	0.0685 ^#^
< 30	95 (76.6)	76 (79.2)	61(70.1)	0.342^$^
≥30	29 (23.4)	20 (20.8)	26 (29.9)	
< 20	4 (3.2)	0 (0.00)	0 (0.00)	0.137^$^
20–29	39 (31.5)	39 (40.6)	29 (33.3)	
30–39	73 (58.9)	51(53.1)	48 (55.2)	
40–49	8 (6.5)	6(6.3)	10 (11.5)	
**Marital status**				
Married	57 (87.7)	46 (93.9)	37 (97.4)	0.183^$^
Not married	8 (12.3)	3 (6.1)	1(2.6)	
**Parity median (IQR)**	1 (0–3)	1 (0–2)	2 (1–4)	0.0253^
0	26 (28.6)	18 (25.4)	11 (17.2)	0.482^$^
1–4	59 (64.8)	50 (70.4)	50 (78.1)	
≥5	6 (6.6)	3 (4.2)	3 (4.7)	
**Gestational age (weeks)**	36 (34–38)	35 (28–39)	37 (34–39)	0.1228^
First trimester	0 (0.0)	3 (7.7)	0(0.0)	0.003*^&^
Second trimester	2 (4.9)	6 (15.4)	0(0.0)	
Third trimester	39(95.1)	30 (76.9)	45 (100.0)	
**Booking status**				
Booked	11(9.1)	3 (4.3)	13 (19.8)	0.003*^$^
Unbooked	110(90.9)	86 (95.7)	59 (80.2)	
**Cadre of Doctor who directly attended to patient**				
Consultant	77 (76.2)	63 (82.9)	46 (67.7)	0.163^&^
Senior Registrar	22 (21.8)	13 (17.1)	19 (27.9)	
Registrar	2 (2.0)	0 (0.0)	3 (4.4)	
**ICU admission**				
Yes	51 (42.5)	18 (19.6)	17 (20.2)	< 0.001*^$^
No	69 (57.5)	74 (80.4)	67 (79.8)	

^#^ *P*-value for ANOVA test. Post-hoc Bonferroni showed: Haemorrhage Vs Sepsis: *P*-value = 0.033; Haemorrhage Vs Hypertension: *P*-value = 0.514; Hypertension Vs Sepsis: *P*-value = 0.492

^$^ P-value for Chi-square test. ^*P*-value for Kruskal Wallis test. ^&^ Fischer’s Exact; * Statistically significant *P*-value < 0.05

## Discussion

This study evaluated the trends, causes and associated factors of maternal mortality at a tertiary health facility over a 13-year period (2007–2019), using Joinpoint regression modelling and other statistical methods.

### Summary of main findings

We found that over the 13-year study period, 483 maternal deaths occurred at an average of 37 deaths per annum. Furthermore, the MMR increased by 4% per annum from 2211 per 100,000 live births in 2007 to 3556 per 100,000 in 2019. Hypertension, haemorrhage, and sepsis contributed about two-thirds of maternal deaths. Anaemia, HIV/AIDS and Sickle cell disease were the leading indirect causes of maternal death.

### Trends in maternal mortality ratio

In contrast to the WHO estimates that suggested that maternal mortality decreased in Nigeria between 1990 and 2015 [2, 9], our study revealed an overall upward trend in maternal mortality of about 4% per annum from 2007 to 2019. Generally, the MMR of between 1700 and 7000 per 100,000 live births in our institution between 2007 and 2019 was very high as compared to the WHO estimate of 800/100,000 live birth for Nigeria [2]. Our finding is similar to that of Idoko et al. from Gambia who found that the decreased MMR trends as reported by WHO for Gambia was at variance with their institutional increasing MMR trends [10]. Likewise, Okonofua et al. found an increased MMR among three secondary health institutions in Lagos state, Nigeria from 2015 to 2016, with similarly high MMR of 1602/100,000 live births [7]. Our centre is also located in same metropolitan city of Lagos, Nigeria. The poor vital registration system in Nigeria continues to be a challenge and may have negatively impacted on the validity of the WHO estimates of maternal mortality in Nigeria and many other LMICs [6].

Furthermore, our institutional MMR was comparable to reports from the northern and Eastern parts of Nigeria [11, 14–17]. However, the MMR at our centre was higher than the reports from Calabar, South-south Nigeria [18] and elsewhere in Nigeria [4]. International comparisons showed that similarly high MMR was also reported from Gambia, Ghana and some LMICs [13, 18]. However, our institutional MMR was about 5 to 100-fold higher than values reported from countries in some part of Africa and among Middle-Income and High-Income Countries [19–23]. Our results are in sharp contrast to reports from HICs that had declining or stable trends of MMR [21–25].

However, some other authors also reported slight decline from some part of Nigeria in line with the WHO report [4, 8, 16–18]. The reported decline may be spurious as these reports generally involved shorter study periods of less than ten years which would make it difficult to draw valid conclusion on trends. Furthermore, these trends studies used descriptive rather than analytical statistics.

The high MMR as highlighted by our study may also be partly due to other reasons. Our centre is a tertiary/ referral institution and would attract a higher proportion of high-risk patients and our findings may not be generally applicable. Being a referral centre however should not be a justification for the high and increasing MMR trends as tertiary hospitals are expected to be appropriately equipped and well-staffed to manage complicated maternal health conditions. Poor investment into health infrastructure and inadequate number of skilled and experienced personnel to handle maternal and child health issues at the primary and secondary healthcare facility could have contributed to the increased MMR at our centre, as the inadequate management of cases at the lower level centres may overburden the tertiary hospitals [3]. Worsening economic outlook, and massive brain drain of health workers continue to accelerate while the effect of the COVID 19 pandemic is yet to be revealed. In order to achieve SDG 3.1 of less than 70 deaths per 100,000 live births by 2030 [26], global MMR must decrease at a rate of 2% Per annum from 2000 to 2030 SDG. For countries in sub-Saharan Africa, the MMR decline must occur at a much higher rate because the starting figures are well above average. Thus, policies geared towards strengthening institutional response to maternal morbidity and mortality are very important in achieving SDG 3.1 [7].

### Trends in causes of maternal mortality

Nearly two-thirds of maternal mortality at our centre was due to hypertensive disorders, sepsis and haemorrhage. This is the usual pattern in most LMICs [11, 14–17]. Thus, efforts geared towards reducing these major causes of maternal mortality will dramatically reduce maternal deaths at our centre. It is noteworthy that the leading causes of maternal mortality in the United Kingdom for instance is sharply different with cardiac disease, pulmonary embolism, epilepsy being the leading contributors [27]. As occurred in LMICs, hypertensive disorders were the leading cause of maternal death at our centre (except for the last three years of the study, 2017–2019) [2, 10, 19]. However, we found that the contribution of hypertensive disorders to overall maternal death in LUTH decreased from 29.9% in 2007–2012 period to 23.6% in 2013–2019 period.

The modest decline in the contributions of hypertensive disorders may be attributable to the use of evidence-based preventive protocols such as the commencement of low dose aspirin and calcium lactate in early pregnancy among high-risk individuals for prevention of preeclampsia [28] and the introduction and implementation of Magnesium Sulphate in the prevention and management of eclampsia [28]. Provision of high-quality antenatal care to all pregnant women for early detection of features of preeclampsia, training of peripheral staff to recognise and manage cases of preeclampsia, seamless referral system to tertiary hospital and strict adherence to protocols for the management of hypertensive disorders can further reduce its contributions to maternal deaths in our environment.

Sepsis has been the second leading cause of maternal mortality at our centre for nearly two decades, contributing about one-quarter of maternal deaths in the last seven years (2013–2019) [3]. However, most studies in LMICs reported sepsis as the third leading cause of maternal mortality after hypertension and haemorrhage [2, 7, 10, 14, 16, 19]. Prevention of prolonged labour, implementation of asepsis, antiseptic and rational antibiotic protocols and policies across various levels of service delivery can considerably reduce maternal deaths due to sepsis [3]. Fake and substandard drugs that engender antibiotic resistance might also have contributed to the risk of septicaemia among maternal deaths [3]. Legislation and enforcement by the relevant authorities will help in mitigating this problem.

During the study period of 2007–2019, haemorrhage was the leading cause of death among the booked patients accounting for one-third of maternal deaths among them. Although haemorrhage was the second leading cause of maternal mortality in most LMICs including Nigeria [10, 11, 14, 15, 19], haemorrhage became the leading cause of death only in the last two years of our study. Furthermore, the contribution of haemorrhage to annual maternal mortality increased from 11% in 2007–2012 to 19.3% in 2013–2019 study period. Thus, the hospital’s strategy in the management of ante-partum and post-partum haemorrhage must be reviewed. Blood banking system must be further improved and high index of suspicion for identifying risks for post-partum haemorrhage must be improved at the centre. Obstetric drills should be conducted regularly to prepare for eventualities. It is not clear why there was no death due to haemorrhage in 2016 even though haemorrhage contributed 15.3 and 15.0% to maternal deaths in 2015 and 2017 respectively.

Obstetric haemorrhage can be acute and unpredictable and may occur even without any identifiable risk factor [29]. As previously noted, haemorrhage may not be the leading cause of maternal deaths among un-booked patients possibly because they might have died before getting to our centre [3].

Indirect causes generally constitute about 25% of maternal death in LMICs [11, 14, 15, 19]. However, indirect causes contributed about 17% of maternal death in our study. Notably, HIV/AIDS contributed about 3.5% between 2007 and 2012 but declined to 2.8% afterwards during the 2013–2019 period. The Prevention of Mother to child Transmission of HIV (PMTCT) program that included the provision of free care (including antiretroviral) and opt-out testing and counselling might have helped to reduce the contribution of HIV/AIDS to maternal mortality. Similar pattern of an initial increase and subsequent drop in HIV attributable maternal deaths was also observed in South Africa – a country with significant HIV burden [30]. However, the PMTCT intervention strategies in Nigeria should be strengthened, and government should increase funding as the foreign donor interventions are dwindling.

Our study indicated a reduced contribution of Sickle cell disease to maternal death from 2.8 to 1.2%. This may be related to the fact that a dedicated unit caring for pregnant patients with sickle cell disease has emerged in our department [31]. Thus, increasing experience and use of evidence-based protocol in the management of such patients might have been responsible for the reduced trends. However, public enlightenment campaign on premarital and prenatal diagnosis and counselling can help prevent the condition and its eventual maternal complications.

### Relationship between socio-biological factors and leading causes of maternal deaths

Between 2007 and 2019, about half of the deceased were in the third decade of life. This is also similar to reports elsewhere in Nigeria and Sub-Saharan Africa [4, 16, 19]. The death of women aged 30 to 40 years would have tremendous impact on family equilibrium and socio-economic productivity [20]. In contrast, some studies reported that women within the ages of 20-30 years constituted most maternal deaths, possibly because of prevalent early marriage in such regions [11, 14, 15].

Although grand multiparous women had a higher risk of maternal death from hypertension and haemorrhage, our study revealed that about 95% of maternal deaths occurred among women with parity less than 5. Thus, all women irrespective of parity should be protected from maternal death.

### Strength and limitations

Although efforts were made during the monthly maternal mortality reviews that were conducted between 2007 and 2019 to clarify and update our entries based on the reviews, some information might have still been missed. One of the strengths of this study is that it is the first to utilise robust Joinpoint trends analysis tool to evaluate maternal mortality trends in Nigeria, to the best of our knowledge. The period of study was also relatively longer than obtains with most other published institutional data on maternal mortality. A longer period of trend analysis usually results in more valid conclusions. Furthermore, inferential statistics were used to improve the understanding of factors affecting maternal mortality. The conclusions from this study may be useful for designing interventions to reduce MMRs in tertiary hospitals that are in the urban cities in Nigeria and similar LMICs. However, the findings from our institutional study may not be generally applicable to the entire Nigerian population with different tiers of private and public health facilities and variations in regional and sociodemographic characteristics.

## Conclusion

There was an annual percentage increase of 4% in MMR from 2007 to 2019. The most recent MMR of over 3000 per 100,000 live births in 2019 is extremely high. The reduction in MMR aimed at achieving SDG 3.1 appears unrealistic if the present trend continues. Hypertensive disorders, sepsis and haemorrhage contributed close to two-thirds of maternal deaths and efforts aimed at preventing death from these conditions can reduce MMR by up to 70% at our centre. Development and implementation of locally relevant protocols for the prevention and management of the leading causes of maternal mortality will go a long way at reducing maternal mortality and making progress towards achieving the sustainable development goal 3.1.

## Supplementary Information

**Additional file 1.** Annual trends in maternal mortality and maternal mortality ratio (2007–2019)

## Data Availability

The data supporting this study are stated in the study. However, the full dataset of the study is available from the corresponding author on reasonable request.

## Abbreviations

APC: Annual Percent Change
HIC: High income countries
ICU: Intensive Care Unit
LMIC: Low- and Middle-income countries
LUTH: Lagos University Teaching Hospital
MDG: Millenium Development Goal
MMR: Maternal Mortality Ratio
PMTCT: Prevention of Mother to Child Transmission of HIV
PPH: Post-partum Haemorrhage
SDG: Sustainable development GoalSSA: Sub-Saharan Africa
WHO: World Health Organisation
