# Supplementary material for: Trends and causes of maternal death at the Lagos University teaching hospital, Lagos, Nigeria (2007–2019)
Source: BMC Pregnancy Childbirth. 2022 Apr 25;22:360. doi: 10.1186/s12884-022-04649-4 (PMC9036837; doi:10.1186/s12884-022-04649-4)
Supplement: Supplementary file 1 — Additional file 1. Annual trends in maternal mortality and maternal mortality ratio (2007–2019) [file 12884_2022_4649_MOESM1_ESM.doc]

**Additional file 1. Annual trends in maternal mortality and maternal mortality ratio (2007 -2019)**

| **Year** | **Number of deliveries** | **Number of live birth** | **Maternal death** | **Maternal mortality ratio (MMR per 100,000 live birth)** |
| --- | --- | --- | --- | --- |
| 2007 | 1905 | 1900 | 42 | 2210.526 |
| 2008 | 1809 | 1753 | 38 | 2167.712 |
| 2009 | 1978 | 1913 | 37 | 1934.135 |
| 2010 | 1828 | 1785 | 46 | 2577.031 |
| 2011 | 2075 | 2028 | 63 | 3106.509 |
| 2012 | 1871 | 1842 | 31 | 1682.953 |
| 2013 | 1654 | 1596 | 49 | 3070.175 |
| 2014 | 695 | 661 | 13 | 1966.717 |
| 2015 | 836 | 756 | 56 | 7407.407 |
| 2016 | 870 | 844 | 31 | 3672.986 |
| 2017 | 1001 | 954 | 21 | 2201.258 |
| 2018 | 783 | 737 | 21 | 2849.389 |
| 2019 | 945 | 900 | 32 | 3555.556 |
